# Editorial: English medium instruction in the Middle East and North Africa

**DOI:** 10.3389/fpsyg.2023.1264435

**Published:** 2023-09-08

**Authors:** Tariq Elyas, Cihat Atar, Samantha Curle, Abu Saleh Mohammad Rafi

**Affiliations:** ^1^Department of Modern Languages and Literature, King Abdulaziz University, Jeddah, Saudi Arabia; ^2^Department of English Language Teaching, Sakarya University, Sakarya, Türkiye; ^3^Department of Education, University of Bath, Bath, United Kingdom; ^4^Department of English and Humanities, University of Liberal Arts, Dhaka, Bangladesh

**Keywords:** Arabicization, EMI, Englishization, internationalization, translanguaging

This Research Topic builds on current EMI work that explores issues related to EMI implementation in higher education institutions, policies, and classroom practices in the MENA region. The Research Topic, thus, is timely as it will provide a range of current investigations that will serve as an important resource for EMI researchers and policymakers at a time when EMI programs, particularly in MENA, are receiving some attention from scholars and policy makers. It also contributes to the shortage of empirically based EMI studies in MENA as the promotion of EMI. The Research Topic sheds light on the current knowledge, policies, and practices of EMI in MENA to pave the way for reforms in policy, conceptual, and empirically informed research recommendations.

Our Research Topic consists of eight original empirical research articles, each addressing an important topic of EMI in MENA. Alwazna explored the use of translation theory through reconciling between Englishization and translanguaging by Arab instructors in EMI higher education classes in training students to be translators. The study findings revealed that most participants used translation theory while training the students, and that they also exercised translanguaging while using translation theory in practical courses. Oraif and Alrashed examined the use of EMI to teach a general course at their university. Their study examined how students' perception about the use of EMI in class. Their study revealed a rather positive attitude toward using EMI in an ESP course in Saudi Arabia. Hence, it was concluded that positive attitudes might not have a significant correlation with positive pedagogical outcomes. The multicultural personality traits of Saudi female EFL undergraduate students is examined by Alghizzi and Alshahrani. The study found that EMI improved cultural empathy and open-mindedness for all participants, while the other dimensions of flexibility, social initiative, and emotional stability improved similarly for only some groups at the academic level, and there was no gradual development or deterioration in these personality traits. Latif and Alhamad's paper reviewed the literature on the following four main pertinent issues: (a) the arguments for and against the Arabicization vs. Englishization of higher education; (b) Arabicization attempts of higher education in the Arab world; (c) current Englishization policies and orientations of Arab higher education systems; and (d) realities of EMI practices in Arab universities. Ntombela examined the potential sociolinguistic problems posed by the hegemony of the English language. Drawing on experiences from the MENA region as well as Eastern and Southern Africa, it was argued that globalization and internationalization work in tandem with neo-colonial and neoliberal operations to create a class of global citizens that must support the economic aspirations of English imperial expansion. Han reported the use of EMI in Tunisia. She utilized ZOOM for teacher trainees reading an academic textbook and posting takeaways on an asynchronous platform over a four-week period. The corpus, comprising 50 journal entries produced by five teacher trainees, was analyzed. Also, Abdeljaoued's article explored students' perspectives on using EMI in Tunisia. His article discussed Tunisian students' attitudes toward EMI, particularly in comparison with French. The study found that students generally had a positive attitude toward English and used a mix of other languages of French, English, and Tunisian Arabic in the classrooms. The Attitude of Iranian students and instructors toward implementing EMI through virtual exchange is investigated by Rakhshandehroo and Rakhshandehroo. Their study explored the attitudes of Iranian students and instructors toward the possibility of implementing EMI in Iran through virtual exchange (VE). It has been identified that the linguistic readiness of students and/or instructors is the primary barrier to the success of EMI through VE in Iran, because EMI is still in its infancy. However, many students showed interest in EMI via VE, primarily to enhance their linguistic skills and global competencies.

Our Research Topic contributes to the discipline by presenting studies that deal with crucial research problems and employ cutting-edge approaches. While it largely focuses on the MENA region, the findings are transferable to related multilingual contexts that uncritically implement EMI, ignoring practical issues such as insufficient English proficiency of students and teachers, which hinders them from effectively engaging with EMI (Rafi and Morgan, 2022). Such implementation caused significant gaps between policies and practices, resulting in different degrees of translanguaging practices. Finally, while EMI is reported to improve cultural empathy and open-mindedness for all participants, it left little room for incorporating non-English languages, thereby creating a nexus of inequity and unfairness in terms of language expectations and hierarchies. In response to the multitude of findings, each article provides recommendations for future research and highlights the implications of EMI in the MENA region.

## Author contributions

TE: Conceptualization, Resources, Supervision, Writing—original draft, Writing—review and editing. CA: Conceptualization, Methodology, Writing—original draft, Writing—review and editing. SC: Conceptualization, Methodology, Supervision, Writing—original draft, Writing—review and editing. AR: Investigation, Methodology, Project administration, Writing—original draft, Writing—review and editing.
